# Dural arteriovenous fistula presenting with recurrent focal status epilepticus and lateralised periodic epileptiform discharges

**DOI:** 10.1007/s10072-024-07325-x

**Published:** 2024-01-19

**Authors:** Isobel Dunbabin, Ramon M. Banez, Aaron de Souza

**Affiliations:** 1 Department of Medicine, Launceston General Hospital, 274-280 Charles Street, Launceston, TAS 7250 Australia; 2 Interventional Neuroradiology Service, Department of Medical Imaging, Royal Hobart Hospital, Hobart, TAS 7000 Australia; 3 Faculty of Medicine, Launceston Clinical School, University of Tasmania, Launceston, TAS 7250 Australia

**Keywords:** Dural arteriovenous fistula, Seizures, Status epilepticus, Aphasia, Lateralised periodic discharges

## Abstract

**Background:**

Dural arteriovenous fistulae (dAVF) are relatively infrequently encountered, and status epilepticus (SE) and lateralised periodic discharges (LPDs) on electroencephalography (EEG) have only rarely been associated with these arteriovenous malformations.

**Methods:**

We present a patient with recurrent presentations with focal SE, aphasia and other focal deficits of cortical function and ictal and peri-ictal LPDs on serial EEG, who was shown to have a left hemispheric dAVF associated with left transverse and sigmoid sinus thrombosis. Seizures proved refractory to four anti-seizure medications but became more amenable to control after successful embolisation of the dAVF, with subsequent resolution of the focal cortical deficits. We discuss the co-occurrence of SE and LPDs with dAVF and review previously reported cases with this rare association.

**Conclusions:**

Our report supports a causative relationship between dAVF and focal SE, manifesting as ictal LPDs on EEG, and highlights the importance of active dAVF management in achieving seizure control. The relatively good patient outcome contrasts to the few similar case reports. Whilst rare, it is important to consider dAVF as a potentially treatable condition underlying new-onset seizures, including SE.

## Introduction

Dural arteriovenous fistulae (dAVF) are relatively rare entities, comprising only 10–15% of intracranial arteriovenous malformations [1], with an estimated incidence of 0.15–0.29 per 100,000 people per year [2, 3]. Status epilepticus (SE) and lateralised periodic discharges (LPDs) are uncommon presentations of this unusual condition and have only been described in a handful of case reports. The outcome of SE due to dAVF is uncertain, with some patients doing well after embolisation and others continuing to have seizures. We present a patient with dAVF associated with cerebral venous sinus thrombosis (CVST) who suffered recurrent focal status epilepticus with electroencephalograms (EEG) demonstrating LPDs, with improved control of seizures after embolisation. Previous reports of dAVF associated with SE and LPDs are discussed.

## Case report

**Fig. 1:**
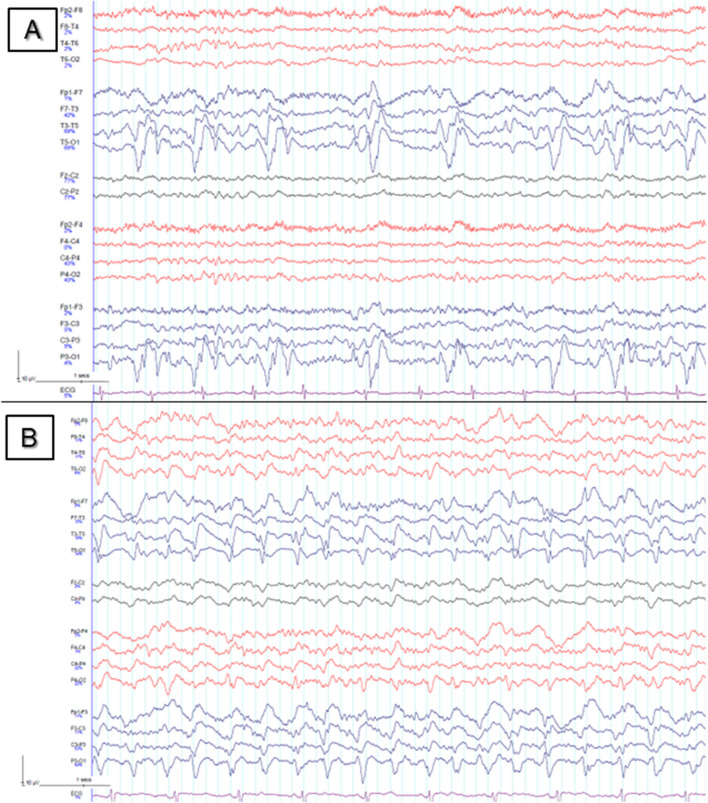
EEG recording at first presentation after treatment of initial generalised convulsive status epilepticus (**A**), showing left temporo-occipital lateralised periodic discharges at 0.8–0.9 Hz, triphasic in morphology, associated with focal spikes. Ictal LPDs are seen in **B** performed after reduction of anti-seizure medications post-embolisation, at 1.5–2 Hz with superimposed spike–wave discharges in the same region. Low-pass filter 30 Hz, high-pass filter 0.5 Hz for both recordings

**Fig. 2:**
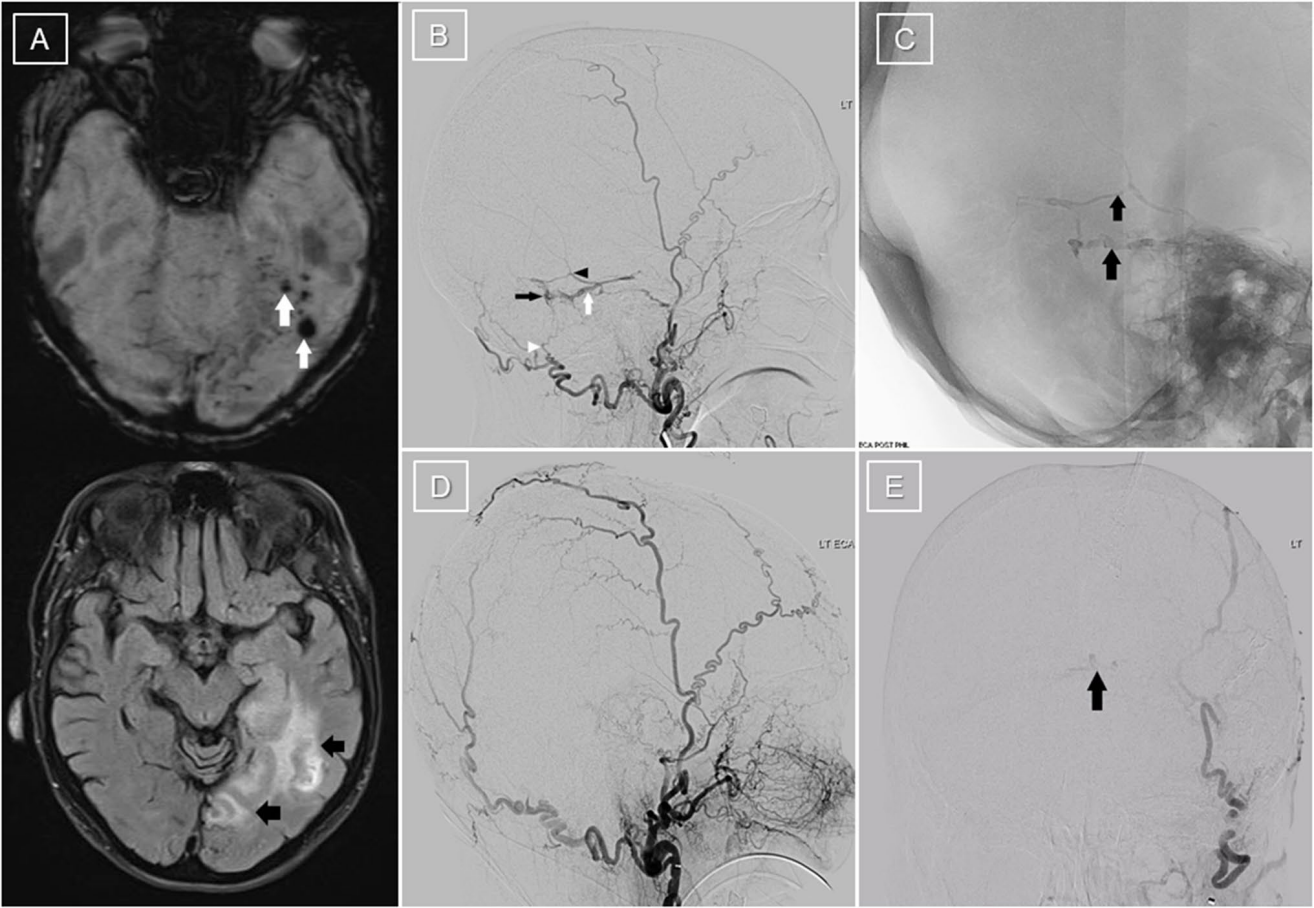
**A** Susceptibility-weighted MRI shows multiple microhaemorrhages in the left temporal and occipital lobes (white arrows), and FLAIR image shows abnormal hyperintensity in the left temporal and occipital lobes (black arrows). **B** Digital subtraction angiography (DSA) with left external carotid artery injection demonstrates dural arteriovenous fistula (dAVF) supplied by branches of the left occipital (white arrowhead) and left middle meningeal (black arrowhead) arteries. Venous drainage is into the left superior petrosal sinus (white arrow), with some reflux into occipital cortical veins (black arrow). **C** Fluoroscopic images following injection of precipitating hydrophobic injectable liquid (PHIL) (MicroVention, Aliso Viejo, CA, USA) via the left middle meningeal and left occipital feeders show embolic material within the fistula (arrows). **D** Post-embolisation DSA shows near-total occlusion of the fistula. Selective injection of the left occipital artery (**E**) demonstrates subtle filling at the confluence of sinuses (arrow) and eventually draining into the transverse sinus, supplied by tiny feeders arising from the left occipital artery. No residual cortical venous reflux

A right-handed 76-year-old woman with pre-morbid ischaemic heart disease, dyslipidaemia, hypertension, degenerative spine disease and polymyalgia rheumatica first presented unconscious to the emergency department at our centre following a prolonged tonic–clonic seizure that was initially unresponsive to two anti-seizure medications (midazolam and levetiracetam). She was found to have a left parietal intraparenchymal haemorrhage, and an EEG demonstrated focal status epilepticus with intermittent bursts of paroxysmal fast activity in the left temporal region rapidly evolving to bilateral polyspike–wave discharges, admixed with rhythmic slowing, LPDs and fast activity over the left hemisphere. Following cessation of clinically obvious seizure activity, she remained obtunded, with EEG demonstrating continuous ictal temporo-occipital LPDs at 0.8–0.9 Hz (Fig. 1A). Magnetic resonance imaging (MRI) showed extensive left temporoparietal gyral oedema, diffusion restriction and punctate microhaemorrhages (Fig. 2A) thought to represent a left temporoparietal venous infarct with haemorrhagic transformation secondary to distal left internal jugular vein and sigmoid sinus thromboses.

After 2 weeks in intensive care, she transitioned to inpatient rehabilitation on oral anticoagulation and maintenance levetiracetam (1.5 g bd), with residual deficits including aphasia, alexia and right homonymous hemianopia. Her aphasia resolved, and she was discharged home, only to represent 3 months later with a sudden-onset mixed expressive and receptive aphasia. MRI demonstrated expected evolution of the venous infarct with no new changes, but an EEG showed left temporo-occipital LPDs. Sodium valproate was added to her regular levetiracetam.

Seven months after initial presentation, she experienced acute confusion with aphasia, ideomotor apraxia, right tactile extinction and homonymous hemianopsia and a Gerstmann syndrome. Further treatment with perampanel and clonazepam was ineffective, and an EEG confirmed the clinical impression of focal status epilepticus whilst on four anti-seizure medications. A lumbar puncture at this time demonstrated normal protein and glucose levels with no pleocytosis. Magnetic resonance angiography and venography demonstrated a possible left-sided dAVF and persisting CVST, and digital subtraction angiography (DSA) demonstrated a left posterior fossa dAVF (Fig. 2B). Embolisation of her left middle meningeal artery and left occipital feeders was successfully performed with very minimal residual shunting post-embolisation (Fig. 2C–E).

Anti-seizure medications were subsequently weaned slowly. Whilst hemianopsia, acalculia, finger agnosia and left–right disorientation improved quickly, persisting fluctuating deficits including alexia, phonemic paraphasia, verbal perseveration, anomia and ideomotor apraxia prompted serial EEG monitoring, with ongoing LPDs and left hemispheric slowing (Fig. 1B). After re-escalating her medication regime, she demonstrated steady improvement on clinical and EEG findings and was able to discharge to an assisted living facility.

## Discussion

We describe a 76-year-old woman presenting with a focal SE, primarily manifesting as aphasia, likely secondary to a left-sided dAVF. Notably, several of her electroencephalograms (EEGs) also demonstrated LPDs, defined as unilateral waveforms with relatively uniform morphology and duration, recurring at nearly regular intervals throughout the entire EEG [4]. We searched online databases including MEDLINE and Google Scholar using the search terms “dural arteriovenous fistula” (or AVF) AND “status epilepticus”, “dural arteriovenous fistula” (or AVF) AND “lateralized periodic discharges” (or LPD) and “dural arteriovenous fistula” (or AVF) AND “periodic lateralized epileptiform discharges” (or PLED). Previously reported cases of dAVF with SE or LPDs or both are presented in Table 1: there have been only a handful of cases reported of focal SE associated with dAVF [5–11], and of these, even fewer demonstrated LPDs on EEG [5, 6].

**Table 1:** Previously reported patients with dural arteriovenous fistulae and status epilepticus or lateralised periodic discharges

Report, year	Age, sex	Seizure type	Other neurological features	EEG	Imaging	Angiographic findings	Outcome	Comment
Current, 2023	76, F	Focal SE: recurrent aphasia evolving to bilateral seizures	Receptive aphasia, anomia, alexia, hemianopia, Gerstmann syndrome	Focal SE with paroxysmal fast activity and LPDs recorded on multiple occasion	CVST involving left TS/Sigmoid sinus; multiple parenchymal microbleeds, left temporal microbleed, venous infarction	Left posterior fossa dAVF, underwent embolisation of left middle meningeal artery and left occipital feeders, very minimal residual shunting	SE partially controlled after dAVF embolisation, needed ongoing treatment with 3 × ASMs	Resolution of focal cortical deficits after dAVF embolisation and seizure control
Dericioglu 2022 [6]	58, M	Right-sided focal seizures of face and hand, evolving to generalised seizures	Dysarthric, aphasic. Prior left hemiparesis and left focal seizures due to right parietal CVST 5 years prior	Moderate background slowing, left fronto-central continuous periodic 1-Hz spike–wave discharges	Restricted diffusion left frontal operculum, insula and perirolandic cortex; sequelae of right parietal venous infarct	Cognard type 4 dAVF	Restricted diffusion improved with AEDs to treat SE. Later EEG demonstrated loss of periodicity and only infrequent left frontal sharp transients	Initial left focal seizures secondary to CVST, but SE from right focal seizures attributed to dAVF. Clinical improvement with ASMs alone, followed by embolisation of dAVF
Quaranta 2021 [8]	82, M	Jerking of right-sided limbs and face, confusion, aphasia	Initial presentation with motor paraparesis and hyperreflexia, after SE developed progressive cognitive deterioration	Slowing, lateralised sharp waves in left parieto-temporo-occipital regions	T2/FLAIR hyperintensities left parietoinsular-occipital regions and left cerebellum, with mass effect from oedema	dAVF left TS. Left IJV occluded	Sudden haemodynamic worsening during procedure led to abandonment. Left ICH 7d later caused death	Spinal and cerebral involvement suggests a tentorial dAVF. Venous congestion and thrombosis led to SE, and venous hypertension implicated in ICH
Tamura 2020 [11]	68, M	Recurrent intractable seizures	–	–	Left basifrontal and basitemporal signal change on FLAIR and ASL	dAVF left anterior cranial fossa draining towards the left mesial temporal lobe	dAVF obliterated after cauterisation of draining vein, SE controlled with medication, resolution of FLAIR and ASL changes	ASMs needed for seizure control after dAVF obliteration, resolution of imaging changes after successful treatment
Derasse 2018 [9]	56, M	Tonic–clonic, “complex-partial” seizures, evolving to refractory SE	Known left carotid-cavernous fistula, after partial embolisation developed dysphagia, hiccup, diplopia, alternating hypoesthesia, right hemiparesis, progressive impairment of cognition	“Diffuse epileptic activity of the left hemisphere”	Following SE: chronic, extensive ischaemic changes in left parieto-occipital white matter	Arterial feeders from left occipital artery, right TS/sigmoid sinus partially occluded. Later, thrombosis of right TS and posterior SSS, venous outflow via left TS and cortical collaterals	Attempted dAVF embolisation produced venous congestion and refractory SE. Progressive occlusion of left TS, right IJV and left innominate veins. Extensive left-sided parenchymal signal change	Clinical worsening attributed to venous ischaemia due to late occlusion of left TS after previous thrombosis of right TS and SSS
Lee 2015 [5]	49, M	Generalised convulsive SE		Bilateral pseudoperiodic lateralised epileptiform discharges		Cognard type 4 dAVF around SSS with narrowing of SSS lumen	Balloon angioplasty of SSS followed by subtotal trans-arterial dAVF embolisation; seizures controlled with ASMs. Left focal SE two months later led to detection of restenosis of SSS and residual dAVF, treated with stenting of SSS and re-embolisation	Seizures recurred after incomplete dAVF treatment, control achieved on two occasions after embolisation, with infrequent focal seizures thereafter
Lee 2015 [5]	71, M	Right hemiparesis, right focal seizures with generalisation		LPDs in the left cerebral hemisphere	Venous infarction, multiple intracerebral hematomas	Cognard type 4 dAVF around left transverse-sigmoid sinus	SE controlled with ASMs. dAVF occluded by trans-venous embolisation; no further seizures	Complete cessation of seizures after treatment of dAVF
Lee 2015 [5]	80, F	Left hemiparesis, altered consciousness		LPDs and episodic lateralised fast activity in the right hemisphere followed by background attenuation		dAVF at right TS, with thrombosis of both transverse and sigmoid sinuses	SE controlled with ASMs. No further seizures after trans-arterial embolisation of the dAVF and stenting of transverse-sigmoid sinuses	Complete cessation of seizures after treatment of dAVF
Moll 2015 [7]	45, F	Focal seizures with generalisation, evolving to SE	Known right carotid-cavernous fistula for 7 years, cognitive deterioration	–	Multiple parenchymal calcifications, dilated TS, CVST left parietal and right temporal. Increased vascular signal on contrast CT	dAVF involving SSS, vertebral artery, cerebellar cortical veins and occipital artery. Right TS occluded distally with collateral flow producing a dilated right superior ophthalmic vein	ASMs, refused embolisation or resection	
Yanagihara 2006 [10]	62, M	SE, semiology not described further	Previous stroke and presumed post-stroke epilepsy, new right hemiparesis	–	Widespread hyperintense signal in the left occipital, parietal temporal cortices	Multiple dAVF associated with superior sagittal sinus; left transverse sinus thrombosis	Cortical signal changes resolved after control of SE and dAVF embolisation	Cytotoxic and vasogenic oedema due to venous hypertension from dAVF and SE may lead to reversible cortical signal changes

*ASL* arterial spin labelling; *ASMs* anti-seizure medications; *CVST* cerebral venous sinus thrombosis; *EEG* electroencephalogram; *FLAIR* fluid-attenuated inversion recovery; *IJV* internal jugular vein; *LPDs* lateralised periodic discharges; *SE* status epilepticus; *SSS* superior sagittal sinus; *TS* transverse sinus

dAVF are pathological connections between the dural arteries and intracranial veins, whether these be venous sinuses, cortical or meningeal veins [1], usually acquired after CVST, trauma, surgery or infection, which can present with varied symptoms determined by both the pattern of venous drainage and the location of the fistula [7, 12]. The likelihood of clinical symptoms due to haemorrhagic and non-haemorrhagic complications is predicted by classification systems such as the Borden and Cognard classification systems which divide dAVF based on the pattern of venous drainage they exhibit on dynamic vascular imaging [1]. The decision to proceed with endovascular treatment in our patient was largely due to the fistula being at risk for haemorrhage (Cognard IIa + b – sinus drainage with cortical venous reflux). The presence of cortical venous drainage makes this an aggressive lesion, reportedly with an annual risk of non-haemorrhagic neurological deficit of 6.9% and an annual risk of intracranial haemorrhage of 8.1% [1]. Because microhaemorrhages were already present, and possibly contributing to her refractory seizures, there was uncertainty as to whether endovascular treatment would improve her symptoms. However, it was still deemed reasonable to treat the fistula, if only to address the cortical venous reflux and therefore decrease the risk of further haemorrhage. At the conclusion of treatment, there was only very minimal dural venous sinus drainage, notably with no cortical reflux (type I pattern, benign).

Whilst there is a strong association between CVST and dAVF, it is unclear in which direction the aetiological relationship lies. CVST may lead to dAVF through venous hypertension causing either the widening of physiological arteriovenous shunts or cerebral hypoperfusion and subsequent neoangiogenesis [1, 5]. Alternatively, turbulent blood flow caused by the dAVF may lead to CVST [5]. Our patient’s CVST persisted after months of anticoagulant therapy with apixaban, and it is unclear in her case whether the persistence of her CVST led to her dAVF or vice versa.

In a series of 53 patients with dAVF, Rabinov et al. reported seizures in three and SE in another, attributed to a left temporal ICH [13]. A recognised but unusual complication of dAVF [5], postulated mechanisms of SE relate to the lesion itself, regional hypoxia due to abnormal shunting of blood flow and venous hypertension resulting in blood–brain barrier disruption and white and grey matter ischaemia due to vasogenic and cytotoxic oedema [5, 7–10, 14]. CVST, if present, can contribute to the venous hypertension [7, 9], which may be alleviated via interventional treatment of dAVF, thereby reducing ischaemia, and thus further seizures and other neurological deficits [9]. As seizures themselves disrupt the blood–brain barrier and potentiate further epileptogenesis, and chronic neural ischaemia carries a poor prognosis, disrupting the epileptogenic cycle through management of the dAVF is key [5, 9, 14]. The clinical utility of this approach has been illustrated in Lee’s case series of patients [5] with SE secondary to dAVF where control of the SE was achieved with a combination of dAVF embolisation and the use of anti-seizure medication (Table 1). Furthermore, in one of these cases, the SE recurred when the first dAVF treatment was incomplete [5], highlighting the crucial role of early dAVF treatment. 93.8% of 48 patients with dAVF-related seizures remained seizure-free after obliteration of their fistulae. Early dAVF treatment, particularly in aggressive cases, was important in achieving seizure freedom [14]. The patients in the table reported by Yanagihara [10], Tamura [11] and Dericioglu [6] demonstrated significant reversibility of grey and white matter imaging changes post-dAVF treatment and SE control, indicating that these imaging findings may be related to cytotoxic and vasogenic oedema secondary to venous hypertension, SE or both [6, 9, 10]. In our patient, angioembolisation of the dAVF did reduce her episodes of focal SE, but she still required anti-epileptic therapy with multiple agents to control her SE. Perhaps recanalisation of her CVST, had this been attempted, may have also improved her symptoms [5, 9]. The control of venous hypertension in this situation would be expected to alleviate neuronal ischaemia, thereby possibly ameliorating clinical outcomes [9].

Patients with focal SE may manifest discrete focal seizures on the EEG (Fig. 1C), typically 30–60 s in duration, or may demonstrate continuous rapid epileptiform discharges without discrete seizures [15]. LPDs, first described by Chatrian in 1964 as “periodic lateralised epileptiform discharges” or PLEDs [16], are still poorly understood and despite a strong correlation with the presence of clinical seizures are the subject of an ongoing debate around their inter-ictal or ictal nature, particularly as they can also appear in the post-ictal period [6, 15, 17]. They occur in a wide range of conditions from cerebrovascular to metabolic disorders [6, 18], and their presence appears to predict a poorer prognosis in terms of morbidity and mortality [19]. The ictal nature of LPDs in focal SE is supported by a periodicity of 1 Hz or greater, by the spatial–temporal evolution of the EEG pattern and by functional neuroimaging changes such hyperperfusion on SPECT and restriction on diffusion-weighted imaging comparable to those seen in status epilepticus [6, 20]. “LPD-plus” are associated with superimposed fast or rhythmic activity or spikes or sharp waves or have a triphasic morphology and are usually peri-ictal or ictal [20]. Clinically, few cases have been described of patients with dAVF experiencing focal status epilepticus (both convulsive and non-convulsive) with LPDs on EEG [5, 6]. Interestingly, there have been several case reports of aphasia ascribed to a focal status epilepticus in association with LPDs [6, 15, 18, 21, 22], as with our patient — although only one related to dAVF [6], underlining the rarity of this association.

In our patient, it is probable that focal status epilepticus, represented electrographically by ictal LPDs, contributed significantly to our patient’s presentation. Whilst aphasia might be ascribed simply to structural damage and neuronal ischaemia arising from her CVST and dAVF and consequent venous hypertension and bleeding, as suggested by a recent case report without convincing epileptiform activity on EEG [23], the temporal variability of our patient’s symptoms and their response to anti-seizure medications suggests that much of her symptomatology was related to seizures. Weaning of our patient’s anti-epileptic medications was attempted, but unfortunately, the episodes recurred, with the patient experiencing episodic aphasic deficits including anomia with phonemic paraphasia. EEG performed several weeks after the embolisation demonstrated ongoing LPDs. Three of the 41 patients with focal SE reported by Drislane et al. had transient but prolonged cognitive deficits, including aphasia, anomia or hemineglect as the clinical manifestations of their seizures [15]. With titration of our patient’s anti-epileptic medications and cognitive rehabilitation, her symptoms and electrographic findings did improve significantly. Even though she did not return to her pre-morbid baseline, we hope that the management has prevented further deterioration.

The table shows that previously reported cases of dAVF manifesting as SE outline poor patient outcomes [8, 9]. Focal SE due to acute cerebrovascular disease results in 67% mortality [15], and the presence of LPDs is generally a poor prognostic indicator in its own right [19, 24]. Nevertheless, it is interesting that the patients in Table 1 similar to ours in which dAVF and LPDs were associated [5, 6] often had, like ours, a relatively good outcome. This conundrum highlights the paucity of published data in this area and, therefore, the importance of case reports such as this to better understand the complex interplay between, and clinical significance of, features such as dAVF and LPDs.

## Conclusions

In conclusion, this case report is significant as one of only a small number of published cases where focal SE and LPDs occur in conjunction with dAVF. It supports a causative relationship between dAVF and focal SE and the importance of active dAVF management in achieving seizure control. LPDs in our patient represented an ictal phenomenon, due to recurrent focal SE, manifesting clinically as recurrent aphasia, apraxia and hemianopia. Finally, the relatively good patient outcome in this case stands in contrast to several of the few similar case reports. Whilst rare, it is important to consider dAVF as a potentially treatable condition underlying new-onset seizures, including SE.

## Data Availability

Data related to this paper are available on request.
